# MRI recovery of the Achilles tendon after percutaneous tenotomy in older children

**DOI:** 10.1186/s13018-021-02407-4

**Published:** 2021-04-13

**Authors:** Manye Yao, Chunxu Zhang, Weyland Cheng, Junhong Guo, Shijie Dong

**Affiliations:** 1 Department of Orthopaedic Surgery, Children’s Hospital Affiliated to Zhengzhou University, Henan Children’s Hospital, Zhengzhou Children’s Hospital, 33 Longhu Waihuan East Road, Zhengzhou, 450018 Henan China; 2 Henan Provincial Key Laboratory of Children’s Genetics and Metabolic Diseases, Children’s Hospital Affiliated to Zhengzhou University, Henan Children’s Hospital, Zhengzhou Children’s Hospital, 33 Longhu Waihuan East Road, Zhengzhou, 450018 Henan China; 3 Department of Radiology, Military Hospital of Henan Province, 18 Jinshui Road, Zhengzhou, 450014 Henan China; 4 Department of Radiology, Children’s Hospital Affiliated to Zhengzhou University, Henan Children’s Hospital, Zhengzhou Children’s Hospital, 33 Longhu Waihuan East Road, Zhengzhou, 450018 Henan China

**Keywords:** Clubfoot, Achilles tenotomy, MRI, Ponseti, Congenital talipes equinovarus

## Abstract

**Background:**

An observational study was conducted to evaluate the recovery of older children with relapsed congenital clubfoot who underwent an Achilles tenotomy for the second time as part of the Ponseti treatment.

**Methods:**

Thirteen patients (19 feet) with congenital clubfoot underwent Achilles tenotomy where magnetic resonance images of the severed tendons were taken after 1, 3, and 6 weeks post-procedure. The participants were categorized into older children who underwent tenotomy for the first time (group A: mean, 4.9±1.8, and range, 2.8–7 years old) and older children who underwent tenotomy for a second time (group B: mean, 4.9±1.5, and range, 3–6.8 years old). The area of high signal intensity between the severed tendons on MRI scans was computed using Python programming language and compared with clinical assessment.

**Results:**

Three weeks after Achilles tenotomy, groups A and B had clinically intact tendons in 9 out of 11 and 2 out of 8 feet, respectively, according to both clinical and MRI assessment. From week 1 to week 3 post-tenotomy, computational analysis showed that the mean high signal intensity area of group A decreased by 88.5±15.2%, which was significantly different (*P* .048 < .05) than the percent reduction of high signal intensity area of group B (69.0±24.9%).

**Conclusion:**

Children who underwent Achilles tenotomy for the second time showed slower tendon recovery on the third week post-procedure. A possible reason for slower healing times may be due to the location of tenotomy in being further away from the musculotendinous junction where extrinsic healing mechanisms take place.

**Supplementary Information:**

The online version contains supplementary material available at 10.1186/s13018-021-02407-4.

## Background

A globally accepted treatment for congenital talipes equinovarus, or clubfoot, is the minimally invasive Ponseti method [1], which first uses a series of five to seven casts over several weeks, gradually correcting the structure of the foot with each new cast. In the last step of correcting the equinus deformity, percutaneous Achilles tenotomy is usually performed in 70–91% of the patients [2] where the Achilles tendon is completely severed using a cataract blade. The final cast is then applied where it is typically removed 3 weeks after tenotomy depending upon the approval of a pediatric orthopedic physician who determines if the severed region is sufficiently full, firm, and continuous, thus indicating the early stages of tendon regeneration.

**Fig. 1:**
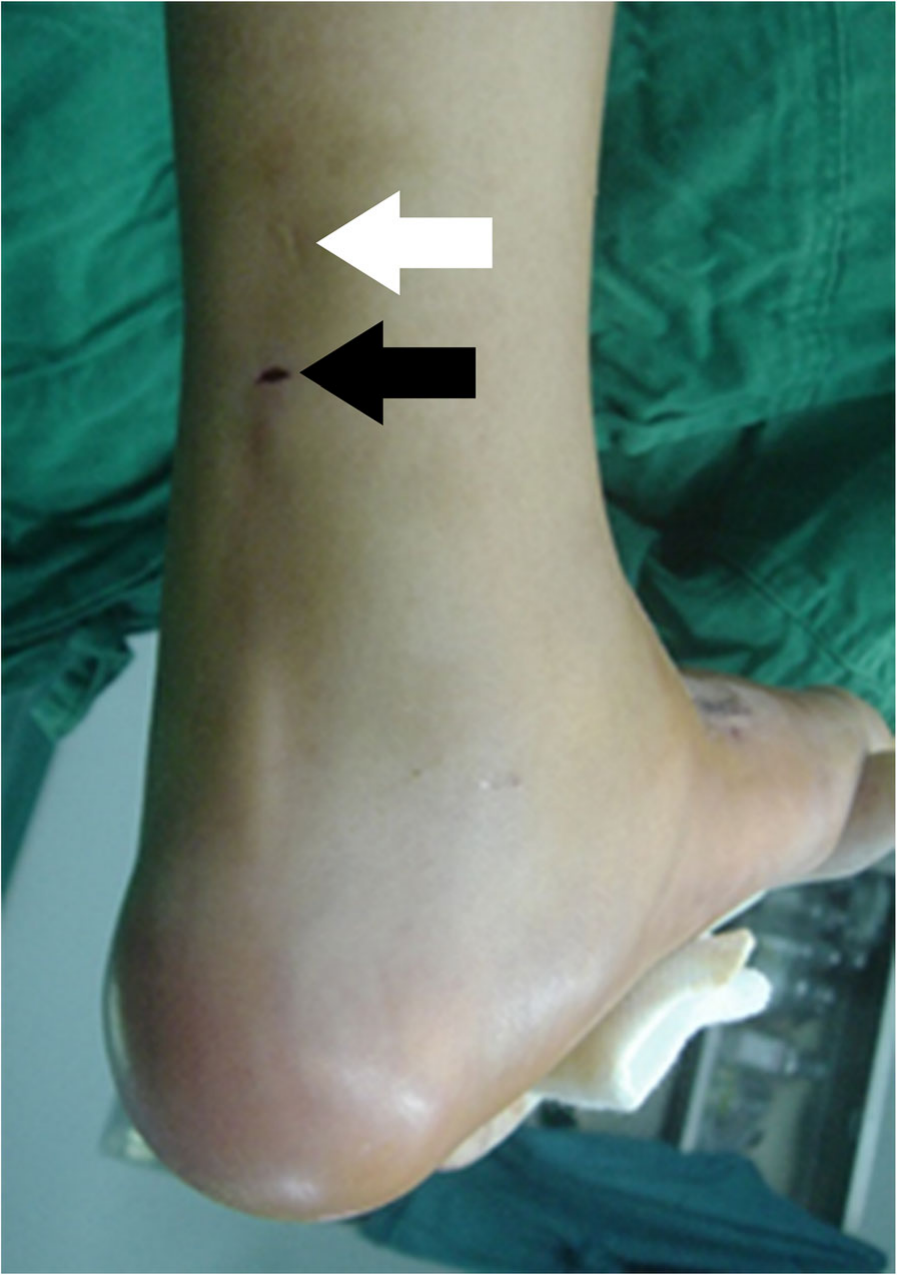
Relapse of clubfoot for this older child led to a second treatment using the Ponseti method and a second Achilles tenotomy procedure. The scar from the first operation (white arrow) is shown to be higher than the incision from the second operation (black arrow)

Clubfoot has a strong tendency to relapse in which case the Ponseti method can be applied again and Achilles tenotomy is performed for a second time [3]. Normally in an Achilles tenotomy procedure, the tendon is transected near the musculotendinous junction where the tendon meets the bulk of the soleus muscle. However, in a secondary procedure for a recurrent case, the tendon can be transected at a location below the first cut (Fig. 1).

In older children, there is controversy about completely severing the Achilles tendon due to the fear of insufficient restoration of tendon continuity. For this reason, a percutaneous lengthening procedure with three cuts has been recommended for children of walking age [4]. However, in the past decade, studies have begun to emerge on performing the Ponseti method on older children [2, 4–9]. These studies have shown that the Ponseti method is still viable in children of ages up to 13 years old.

As of known to the authors, there are currently no comparative research studies that have used imaging modalities to examine the early recovery of the Achilles tendon after tenotomy in older children with recurrent clubfoot. This investigation uses magnetic resonance imaging (MRI) to compare the recovery of older children who have undergone Achilles tenotomy for the first and second time.

## Methods

### Participants

Data from April 2012 to July 2013 was collected, consisting of clinical assessments and MRIs of 13 older children (19 feet in total) with congenital and residual equinus deformity who underwent Achilles tenotomy surgery followed by plaster casting immobilization. The patients were examined and treated in Children’s Hospital Affiliated to Zhengzhou University and MRI scans were conducted at Military Hospital of Henan Province. Informed consent of the parents or guardian of each patient was obtained and use of this data was reviewed and approved by the hospital’s ethic review board committee.

Older children who received percutaneous Achilles tendon severance for the first and second time were classified into group A and group B, respectively. Group A consisted of six participants (11 feet) with a mean age of 4.9 ± 1.8 (range, 2.8–7) years old. Group B consisted of seven participants (eight feet) with a mean age of 4.9 ± 1.5 (range, 3–6.8 years old).

### Data collection

In accordance with past studies, a duration of 3 and 6 weeks post-tenotomy was used as main assessment periods [10]. 3D MRI scans (Magnetom Essenza 1.5T MRI System, Siemens, Berlin, Germany) of the operated Achilles tendons were conducted on groups A and B on the first, third, and sixth week postoperation. MRI scans were conducted for qualitative evaluation with the following conditions: sagittal t1-weighted, TR/TE, 350.0/11.0; sagittal t2-weighted, TR/TE, 2300.0/64.0; and sagittal proton density (PD) weighted, TR/TE, 2380.0/24.0. The sagittal PD-weighted MR images were also used for quantitative analysis.

### Qualitative analysis

One week after the Achilles tenotomy, MRI was used to determine if the tendon was completely severed. Three weeks after tenotomy, following an MRI examination, the cast was removed. A pediatric orthopedist examined the patient’s Achilles tendon while the patient was performing plantar flexion movements of the ankle joint to assess whether tendon recovery was adequate to discontinue immobilization. The tendons were evaluated to be clinical intact by assessing their firmness, fullness, and continuity. The patients were then fixed with plaster casts. At week 6, the patients were again evaluated through plantar flexion movements to check for the clinical intactness of the tendon. MRI scans were used as a comparative evaluation method. In T2-weighted images, tendon had low signal intensities to black, muscle had intermediate signal intensities, and fluid had high signal intensities.

### Quantitative analysis

**Fig. 2:**
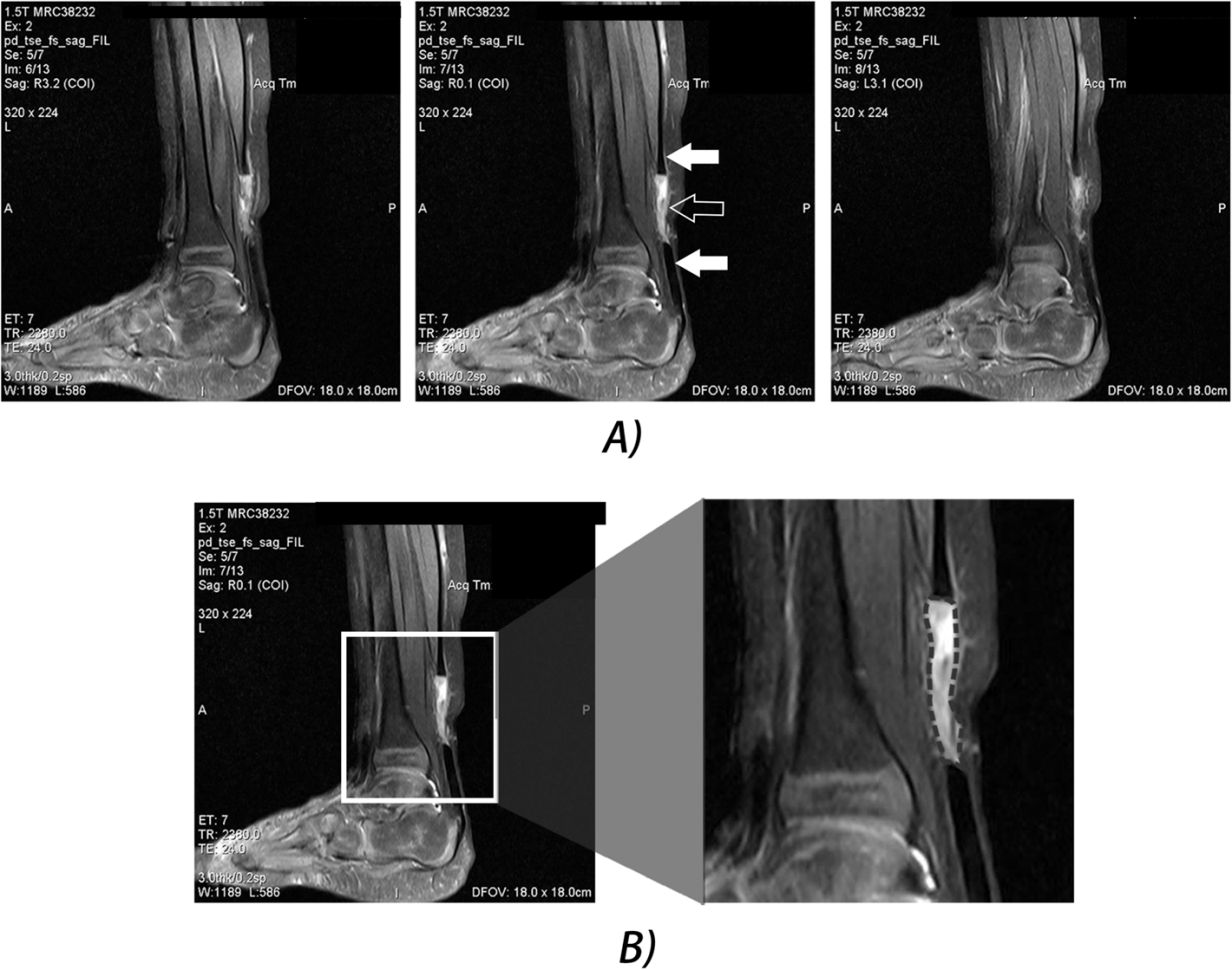
Example case of a sagittal PD-weighted MR image (TR/TE, 2380.0/24.0) where the area between the severed tendons was cropped. **a** From successive MR images, the middle image was chosen to represent the center of the tendon with respect to the sagittal cross-section. The middle image shows the severance of the tendon (white arrow) and the tendon gap (outlined arrow) with higher signal intensities than the surrounding muscle and tendons. **b** The area between the severed tendons was manually cropped and the pixel count of high signal intensity were computed in Python

MRI scans were used to determine the distance between the severed tendons at week 1. In order to quantify the tendon’s recovery, sagittal PD-weighted MR images (TR/TE, 2380.0/24.0) were used to determine the area of high signal intensity between the severed tendons on weeks 1, 3, and 6. For each week and treated foot, the MR image depicting the center of the sagittal cross-sectional area was chosen to determine the quantity of high signal intensity within the tendon gap (Fig. 2a). Based on the opinion of an MRI specialist, the area of interest in the tendon gap was manually cropped from the images (Fig. 2b). A customized Python script was used to compute the total area of high signal intensity (pixels) within the cropped image. High signal intensity pixels were determined based on grayscale values (>190, range 0–255) and the accuracy of the filtered high signal intensity regions was also confirmed by an MRI specialist. The Mann-Whitney *U* test was used to compare the statistical significance between the high signal intensities of the two groups as well as their percent reduction from weeks 1 to 3 and weeks 1 to 6.

## Results

### Qualitative analysis

Results for the qualitative analysis may be seen in Table 1. One week after Achilles tenotomy, the tendon was confirmed to be detached in all groups by observation of the MRI scans.

**Table 1:** Comparison between clinical and MRI evaluation of tendon recovery

	Week 3	Week 6
**Group A**		
**Number of tendons clinically intact**	9/11	11/11
**Number of tendons shown to have continuity by MRI**	9/11	11/11
**Group B**		
**Number of tendons clinically intact**	2/8	8/8
**Number of tendons shown to have continuity by MRI**	2/8	7/8

At 3 weeks post-tenotomy, the continuity, texture, and fullness of the Achilles tendon were examined after cast removal during ankle joint plantar flexion movements. In group A, the Achilles tendons were clinically determined to be clinically intact in nine out of eleven feet. MRI evaluation revealed that nine out of eleven feet also showed adequate tendon recovery. In group B, most of the Achilles tendons were not full during plantar flexion. Two out of eight feet were clinically assessed to be sufficiently intact by week 3. MRI evaluation revealed that two out of eight feet showed adequate recovery.

**Fig. 3:**
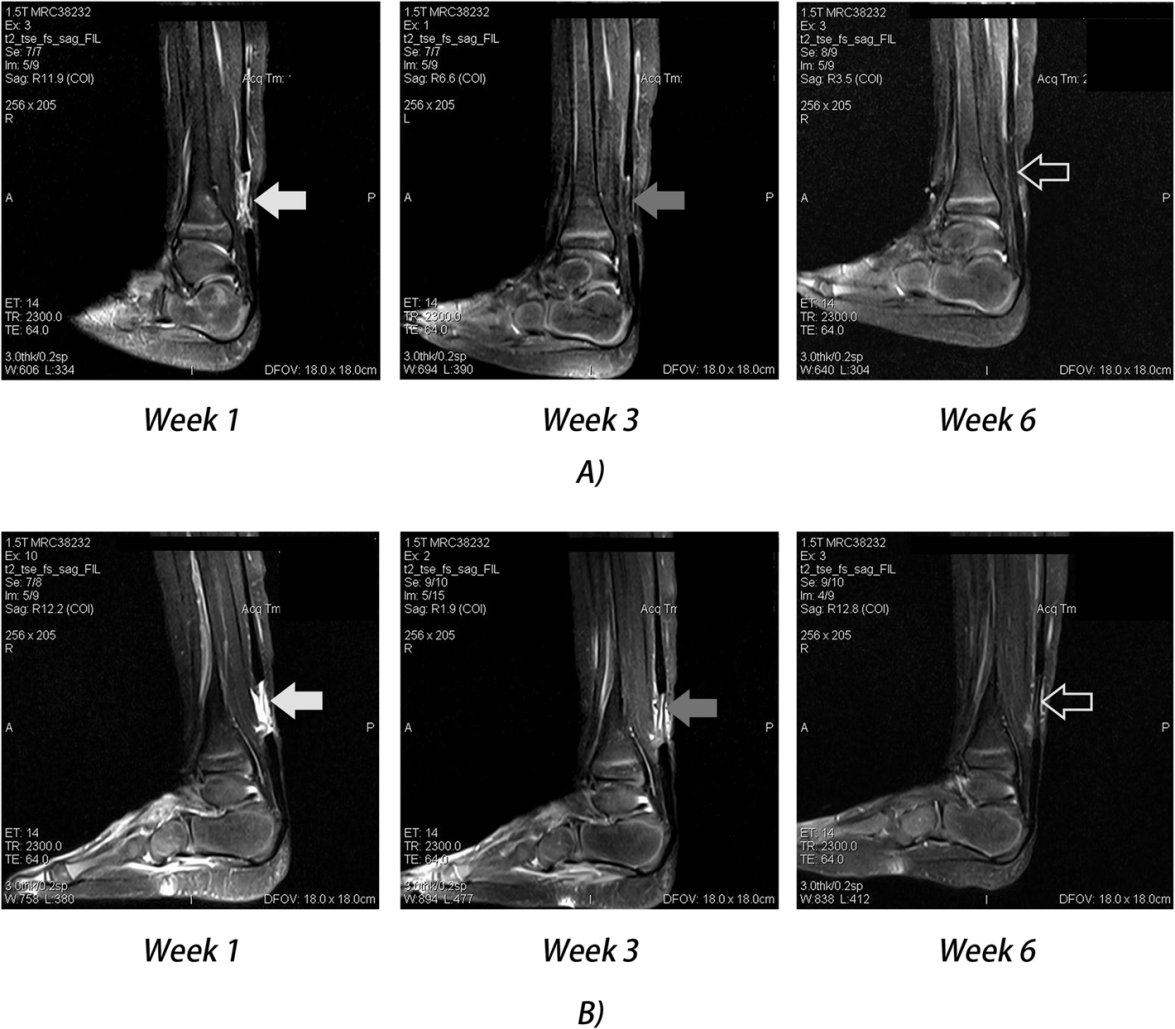
MRI scans (sagittal T2-weighted, TR/TE, 2300.0/64.0) of two patients 1-, 3-, and 6 weeks post-tenotomy. **a** Patient from group A showed separation of the Achilles tendon 1 week after surgery (white arrow). Week 3 showed low and low-intermediate signal intensities in the tendon gap where the early period of tendon regeneration can be seen (gray arrow). Week 6 showed the low intensity signal of the regenerated tendon (outlined arrow). **b** Patient from group B showed separation of the Achilles tendon 1 week after surgery (white arrow). Week 3 showed high signal intensities in the tendon gap, yet tendon regeneration can be seen in the two vertical, low-intermediate signal intensities (gray arrow). Week 6 showed the low intensity signal of the regenerated tendon (outlined arrow)

On week 6 post-tenotomy, groups A and B were once again evaluated after cast removal and all operated tendons were determined to be clinically intact. MRI observation showed that one patient in group B had insufficient recovery on the sixth week. An example of the MRI scans may be seen in Fig. 3.

### Quantitative analysis

The distance measured between severed tendons 1 week after the Achilles tendon procedure was 2.69 ± 0.83 (range, 1.66–4.21) cm and 2.18 ± 0.61 (range, 1.34–2.97) cm for groups A and B, respectively.

Computational analysis revealed that group B had a larger average area of high signal intensity in all 3 weeks with a mean area of 731 ± 432, 223 ± 197 and 16 ± 45 pixels on weeks 1, 3, and 6, respectively. Group A had a mean high signal intensity of 440 ± 176, 49 ± 69, and 0 ± 0 pixels on weeks 1, 3, and 6, respectively.

For weeks 1 and 6 post-tenotomy, groups A and B had no significant difference in their mean area of high signal intensity with a *P*-value of .099 (>.05) and .241 (>.05), respectively. For week 3, group A vs B (P = .037 < .05) had a statistically significant difference in the amount of high signal intensity, meaning that the null hypothesis of having an equal mean should be rejected.

From week 1 to week 3, group A and B’s mean percent reduction in high signal intensity area was 88.5 ± 15.2% and 69.0 ± 24.9%, respectively. These two values were significantly different with a *P*-value of .048 (<.05). From week 1 to week 6, group A and B’s mean percent reduction in high signal intensity area was 100 ± 0.0% and 93.1 ± 19.4%, respectively. These two values were not significantly different with a *P*-value of .264 (>.05).

## Discussion

There are two contradictory theories on the recovery of tendon, which consists of extrinsic and intrinsic healing. The theory regarding extrinsic healing suggests that the tendon has no internal ability to heal itself, so adhesion formation is necessary by means of inflammatory cells and fibroblasts in the blood supply that permeate through extraneous tendon vasculature. In contrast, intrinsic healing suggests that regeneration of the muscle tendon lacks adhesion formation, rather it stems from the proliferation of epitenon cells and endotenon cells derived from the blood supply within the tendon [11]. In actuality, the recovery of the tendon may require the combination of both internal and external healing processes [12].

Clinical evidence suggests that the broken ends of a severed Achilles tendon can recover completely and some experts have expressed doubt about completely severing the tendon for fear that it would cause irreparable damage. In 2006, Barker and Lavy used ultrasound images to show the recovery of patients with a mean age of 127 days who had undergone Achilles tenotomy surgery [10]. Three weeks after tenotomy, they determined that 8 out of 11 tendons were clinically intact and that 10 out of 11 tendons were shown to have continuity by ultrasound. Maranho et al. [13] used ultrasonography to conclude that a predominantly intrinsic repair mechanism was occurring where the gap between the severed tendons was being filled by hypoechoic tissue by the third week post-tenotomy. A study by Agarwal et al. [5] used ultrasonography to reaffirm the functional continuity of the Achilles tendon 4 weeks post-tenotomy. Niki et al. [9] examined the tendon gap for 2 years post-tenotomy to observe its healing process with ultrasound images. Despite these studies, Nasr et al. [14] have indicated that routine ultrasound evaluations conducted intraoperatively or postoperatively provide no value towards clinical assessments.

In terms of children with recurrent clubfoot, a comparative study by Khan et al. [15] evaluated the effectiveness of the Ponseti method for infants with recurrent clubfeet in comparison to infants with virgin clubfeet. They found no statistical differences when comparing the number of casts used as well as in the improvement of the Pirani score. Agarwal et al. [16] conducted a functional study for the recovery time of older children who underwent Achilles tenotomy. The recovery time of older children (11–13 years old), younger children (2–5 years old), and children with a history of previous treatment were compared. They concluded that older children and children with recurrent clubfoot required a longer recovery period.

In this study, there was no relationship found between the area of high signal intensity and the distance of the tendon gap measured in week 1. Results indicated that older children with relapsed clubfoot who underwent Achilles tenotomy for a second time were not ready at week 3 and required a longer recovery period before the removal of the final cast during the Ponseti method. On the other hand, most of the children in group A were deemed to have clinically intact Achilles tendons at week 3, which was consistent with literature.

The computed mean high signal intensity between the severed tendons of group B showed that not all participants had adequate muscle and tendon recovery. The MR images in week 3 showed the regrowth of the tendon, evident in the vertical and linear extensions of the intermediate signal intensity in the tendon gap. Results indicated that group B could be assessed for cast removal by week 6.

The slower recovery rate of group B could be due to the position at which the tendon was severed. There are three blood resources of Achilles tendon: the neighboring muscular branches, arteries in the connective tissue surrounding the Achilles tendon, and blood vessels in the periosteum where the tendon adheres to the bone. As group A had tenotomy performed closer to the calf muscle, additional supply of blood was available from the musculotendinous junction, resulting in an increased source of extrinsic healing and a faster recovery rate. Whereas Maranho et al. [13] reported that the recovery of the tendon was predominantly intrinsic, our findings lead us to believe that extrinsic healing is still a large factor in the tendon’s recovery rate.

Limitations in this study include the limited number of patients who underwent MRI examination resulting in a slightly low statistical power when comparing the clinical intactness of the tendons. Week 3 sample size calculations determine that a total of 22 feet would have been preferable for sufficient power in clinical assessments. Based on quantitative high-intensity signal differences in week 3, a sample size of five would have been sufficient for adequate power. MRI is known to be an effective method for clinically evaluating tendons [17]; however, we generated a computational method to quantitatively and objectively evaluate signal intensity. Clinical evaluation of the intactness of the tendons was subjective, although all assessments were conducted by one orthopedic physician. Due to the young age of a portion of participants, tip-toes walking was not used as a comparative method.

## Conclusion

In this study, we compared the early recovery period of older children with clubfoot or recurrent clubfoot who underwent an Achilles tenotomy operation for the first and second time, respectively. Based on clinical assessment and MRI analysis, most children who underwent Achilles tenotomy for the first time were sufficiently healed to have their cast removed at 3 weeks post-operation. Comparatively, six out of eight children who underwent Achilles tenotomy for the second time were not sufficiently healed to discontinue cast immobilization at 3 weeks post-operation. Rather, the operated tendon of children with recurrent clubfoot was found to be intact on the sixth week post-operation. In recurrent cases, the tendon was severed at a position lower from the first cut to avoid improper healing at the previously scarred region, rendering the second tenotomy further away from the musculotendinous junction. The difference in the location of tenotomy could be a reason for differences in recovery rate and an indication towards the role of extrinsic healing mechanisms derived from inflammatory cells and fibroblasts in the blood supply. Therefore, timing for the final cast removal under the Ponseti method should take into consideration the slower rate of recovery of older children undergoing Achilles tenotomy for the second time.

## Supplementary Information

**Additional file 1:.** Supplementary data.

## Data Availability

Tabulated data is available in supplementary materials.
